# The Interplay between Perioperative Oxidative Stress and Hepatic Dysfunction after Human Liver Resection: A Prospective Observational Pilot Study

**DOI:** 10.3390/antiox13050590

**Published:** 2024-05-11

**Authors:** Florian Primavesi, Thomas Senoner, Sophie Schindler, Aleksandar Nikolajevic, Pietro Di Fazio, Georg Csukovich, Silvia Eller, Bettina Neumayer, Markus Anliker, Eva Braunwarth, Rupert Oberhuber, Thomas Resch, Manuel Maglione, Benno Cardini, Thomas Niederwieser, Silvia Gasteiger, Eckhard Klieser, Herbert Tilg, Stefan Schneeberger, Daniel Neureiter, Dietmar Öfner, Jakob Troppmair, Stefan Stättner

**Affiliations:** 1 Department of Visceral, Transplant and Thoracic Surgery, Medical University of Innsbruck, 6020 Innsbruck, Austria; sophie.schindler@srrws.ch (S.S.); eva.braunwarth@tirol-kliniken.at (E.B.); rupert.oberhuber@i-med.ac.at (R.O.); thomas.resch@i-med.ac.at (T.R.); manuel.maglione@i-med.ac.at (M.M.); benno.cardini@i-med.ac.at (B.C.); silvia.gasteiger@i-med.ac.at (S.G.); stefan.schneeberger@i-med.ac.at (S.S.); dietmar.oefner@i-med.ac.at (D.Ö.); 2 Daniel Swarovski Research Laboratory, Department of Visceral, Transplant and Thoracic Surgery, Medical University of Innsbruck, 6020 Innsbruck, Austria; aleksandar.nikolajevic@i-med.ac.at (A.N.); silvia.eller@i-med.ac.at (S.E.); jakob.troppmair@i-med.ac.at (J.T.); 3 Department of General, Visceral and Vascular Surgery, Salzkammergutklinikum, 4840 Vöcklabruck, Austria; stefan.staettner@ooeg.at; 4 Department of Anaesthesiology and Intensive Care Medicine, Medical University of Innsbruck, 6020 Innsbruck, Austria; thomas.senoner@i-med.ac.at; 5 Department of Visceral, Thoracic and Vascular Surgery, Philipps-Universität Marburg, 35043 Marburg, Germany; difazio@med.uni-marburg.de; 6 Small Animal Internal Medicine, Vetmeduni, 1210 Vienna, Austria; 7 Institute of Pathology, Paracelsus Medical University/University Hospital Salzburg (SALK), 5020 Salzburg, Austria; b.neumayer@salk.at (B.N.); e.klieser@salk.at (E.K.); d.neureiter@salk.at (D.N.); 8 Central Institute of Medical and Chemical Laboratory Diagnostics, Medical University of Innsbruck, 6020 Innsbruck, Austria; markus.anliker@tirol-kliniken.at; 9 Department of Internal Medicine I, Gastroenterology, Hepatology, Endocrinology and Metabolism, Medical University of Innsbruck, 6020 Innsbruck, Austria; herbert.tilg@i-med.ac.at

**Keywords:** oxidative stress, liver resection, liver dysfunction, post-hepatectomy liver failure, outcome

## Abstract

Post-hepatectomy liver failure (PHLF) remains the major contributor to death after liver resection. Oxidative stress is associated with postoperative complications, but its impact on liver function is unclear. This first in-human, prospective, single-center, observational pilot study evaluated perioperative oxidative stress and PHLF according to the ISGLS (International Study Group for Liver Surgery). Serum 8-isoprostane, 4-hydroxynonenal (4-HNE), total antioxidative capacity, vitamins A and E, and intraoperative, sequential hepatic tissue 4-HNE and UCP2 (uncoupling protein 2) immunohistochemistry (IHC) were assessed. The interaction with known risk factors for PHLF and the predictive potential of oxidative stress markers were analyzed. Overall, 52 patients were included (69.2% major liver resection). Thirteen patients (25%) experienced PHLF, a major factor for 90-day mortality (23% vs. 0%; *p* = 0.013). Post-resection, pro-oxidative 8-isoprostane significantly increased (*p* = 0.038), while 4-HNE declined immediately (*p* < 0.001). Antioxidative markers showed patterns of consumption starting post-resection (*p* < 0.001). Liver tissue oxidative stress increased stepwise from biopsies taken after laparotomy to post-resection in situ liver and resection specimens (all *p* < 0.001). Cholangiocarcinoma patients demonstrated significantly higher serum and tissue oxidative stress levels at various timepoints, with consistently higher preoperative values in advanced tumor stages. Combining intraoperative, post-resection 4-HNE serum levels and in situ IHC early predicted PHLF with an AUC of 0.855 (63.6% vs. 0%; *p* < 0.001). This was also associated with grade B/C PHLF (36.4% vs. 0%; *p* = 0.021) and 90-day mortality (18.2% vs. 0%; *p* = 0.036). In conclusion, distinct patterns of perioperative oxidative stress levels occur in patients with liver dysfunction. Combining intraoperative serum and liver tissue markers predicts subsequent PHLF. Cholangiocarcinoma patients demonstrated pronounced systemic and hepatic oxidative stress, with increasing levels in advanced tumor stages, thus representing a worthwhile target for future exploratory and therapeutic studies.

## 1. Introduction

Reactive oxygen species (ROS) are important contributors to physiological cell signaling but are also involved in disease development processes and arise during surgical interventions [1,2,3,4]. Especially after complex, extensive surgery or in elderly patients, ROS can add to cellular stress and organ damage [5,6,7,8]. Moreover, different levels of oxidative stress were reported depending on the surgical access used (open vs. minimally invasive) and the type of anesthesia applied [9,10]. On top of increased baseline ROS states in patients with chronic organ damage or cancer, [11,12] significant acute oxidative stress due to inflammation, blood loss, and ischemia can result in complications and impair patient recovery, with a potential impact on oncological outcomes [5,13,14,15,16,17]. In the field of liver resection, in contrast to liver transplantation, only limited evidence exists on the perioperative dynamics of oxidative stress serum markers and their association with postoperative outcomes [5,12,13,14,18,19].

While perioperative innovations have helped to push borders in hepatobiliary surgery, resections are still mainly limited by the function and volume of the liver remaining in situ, referred to as the future liver remnant (FLR) [20,21,22]. In the case of an insufficient FLR, potentially life-threatening postoperative liver dysfunction (“post-hepatectomy liver failure”/PHLF) can occur, often associated with secondary co-factors such as infections or bleeding. The International Study Group of Liver Surgery (ISGLS) defines PHLF as bilirubin and prothrombin time outside the normal range on postoperative day (POD) 5 or later, further classifying clinical severity grades A, B, and C [23]. While reported rates in the literature range between 8 and 12% [22,23,24], these increase to over 30% depending on the indication, extent of resection, and pre-existing liver parenchyma pathology, such as steatosis or cirrhosis [25,26,27]. The importance of PHLF as a major contributor to all-cause mortality is underlined by data showing that up to 70% of patients who die after liver resection fulfill the criteria for PHLF and half of these cases die as a direct consequence of PHLF [20,23,25,27,28]. Consequently, mortality rates of 15% are reported in clinically challenging indications, such as perihilar cholangiocarcinoma [29]. Due to limited therapeutic options with a lack of efficient liver support devices, prevention and early anticipation of imminent PHLF are paramount [22,26,27,30,31].

The association between perioperative oxidative stress and hepatic function after liver resection remains unclear. A few publications have examined the connection with overall postoperative outcomes [5,13,14], but no study has so far assessed a direct link with pre-defined PHLF. Therefore, the aim of this pilot study was to investigate the perioperative dynamics of systemic and hepatic oxidative stress levels, their association with different patient-, disease-, and surgery-related factors, and a potential connection with PHLF. These first in-human data could help to design larger multicenter validation studies and, ultimately, plan therapeutic randomized controlled trials to prevent PHLF.

## 2. Materials and Methods

This prospective, single-center, observational pilot study recruited consecutive patients undergoing elective liver resection for benign or malignant liver tumors from June 2017 to December 2018 at the Department of Visceral, Transplant, and Thoracic Surgery at the Medical University of Innsbruck. Exclusion criteria included age under 18 years, pregnancy, non-elective indication for liver resection, such as trauma or abscess, or cases with simultaneous substantial extrahepatic multiorgan resection (e.g., pancreatectomy, gastrectomy). In the case of planned two-stage resections, only the second (major) resection was included. Written informed consent was obtained from all patients according to the study protocol, as previously approved by the local ethics committee (EC number AN2017-0035370/4.13; 05/04/2017, accessed on 1 April 2024).

The primary endpoint comprised the assessment of the perioperative dynamics of serological and histopathological tissue markers of oxidative stress in this cohort of hepatectomy patients. Secondly, patients were stratified by the subsequent occurrence of PHLF according to the ISGLS criteria [23] to detect differences in systemic and local hepatic oxidative stress levels between these two subgroups. In this context, the association with overall complications and mortality was also assessed. Thirdly, the study investigated co-factors influencing oxidative stress levels acutely (e.g., the extent and technique of resection, application of inflow control, type of surgical access, etc.) and chronically (e.g., underlying liver disease or indication for surgery). The study protocol was previously published as part of an MD diploma thesis (T.S.) at the Medical University of Innsbruck (Verbund-ID-Nr: AC15228130; https://bibsearch.uibk.ac.at/AC15228130, accessed on 1 April 2024).

### 2.1. Surgical and Anesthesiologic Details

All patients underwent open liver resection through a midline incision and optional additional right transverse extension. The application of inflow occlusion (the Pringle maneuver) was used at the main surgeon’s discretion and usually applied in an intermittent fashion, starting with 10 min of clamping as preconditioning, followed by 5-min breaks alternating with 15 min of clamping. Parenchymal transection was undertaken with the Kelly clamp–crush technique or a cavitron ultrasonic surgical aspirator (CUSA) device, complemented by ligatures, clips, and stapling devices for vascular and biliary structures. An isoflurane- and propofol-based balanced anesthesia was used for all patients. Major liver surgery was defined as the resection of >3 anatomical segments or >6 non-anatomical resections and ablations [32,33]. Postoperative mortality was recorded as death within 90 days. Postoperative morbidity was defined as any deviation from the normal postoperative course within 90 days, graded according to the Clavien–Dindo classification (grades I-II: mild complications; grades IIIa-V: severe complications).

### 2.2. Sample Collection and Analysis

Peripheral or central venous whole blood samples were collected preoperatively before the induction of anesthesia intraoperatively after the completion of the hepatic resection phase and on the morning of the first and fifth postoperative days (POD1 and POD5). Routine blood markers were evaluated through the hospital central laboratory comprising thrombocytes, glutamic–oxaloacetic transaminase (GOT/AST), glutamate–pyruvate transaminase (GPT/ALT), C-reactive protein (CRP), lactate dehydrogenase (LDH), lactate, bilirubin, albumin, and prothrombin time (PT). IL-6, IL-8, IL-10, and TNF-α were analyzed in the hospital’s certified rheumatology lab using high-sensitivity magnetic Luminex^®^ performance assays (flexible bead-based multiplex assay; R&D Systems, Inc., Minneapolis, MN, USA). Additional serum sample tubes were collected at all timepoints, allowed to clot for 20 min, and then centrifuged at 3000 G for 10 min. The supernatant was immediately aliquoted into Eppendorf tubes and stored at −80 °C for later analysis of the oxidative stress markers.

Non-tumoral histological liver samples were taken at two different timepoints: Immediately after the laparotomy, one liver punch biopsy (4 mm) was taken from the right and left liver. Immediately after the hepatic transection phase, another punch biopsy was taken from the FLR remaining in situ, as well as the resected specimen. Laboratory researchers and pathologists examining the oxidative serum and tissue markers were blinded regarding the patient characteristics and clinical outcomes.

### 2.3. Markers of Oxidative Stress

Pro-oxidative stress markers included 8-isoprostane (8-iso Prostaglandin F2α, a product of tissue phospholipid oxidation by ROS) and 4-hydroxynonenal (4-HNE, a product of lipid peroxidation), while the antioxidative markers comprised vitamins A and E, as well as the total antioxidative capacity (TAC). These markers were chosen based on the previously published literature showing significant associations with tissue damage, organ dysfunction, inflammatory complications, aging, malignant diseases, and surgical procedures [5,10,34,35,36].

8-isoprostane serum levels were measured using an 8-isoprostane ELISA Kit (516351; Cayman Chemical, Ann Arbor, MI, USA) according to the manufacturer’s manual. In this competitive, enzymatic, spectrophotometric assay, the free 8-isoprostane serum fraction binds to rabbit IgG mouse monoclonal antibodies pre-coated on wells, and an 8-isoprostane-acetylcholinesterase conjugate was used as a tracer for quantification. 4-HNE serum levels were determined using a human 4-HNE ELISA Kit (MBS706111; Mybiosource, Inc., San Diego, CA, USA), a competitive inhibition enzyme immunoassay. Serum samples were diluted 1:200 with the included sample diluent. Standards were then added to a pre-coated microtiter plate with HNE-specific antibodies along with Horseradish peroxidase (HRP)-conjugated HNE, resulting in a competitive inhibition reaction. A substrate solution was added to produce a color reaction, which develops inversely with the 4-HNE concentration in the sample.

For the TAC assay kit (MAK187; Sigma-Aldrich, Inc., St. Louis, MO, USA), no protein mask was applied to measure both the antioxidant capacity of small molecules and proteins. The antioxidant capacity was determined based on the reduction of Cu2+ to Cu+, which was then chelated with a colorimetric probe. Trolox, a water-soluble Vitamin E equivalent known for its antioxidant properties, functioned as the standard for the TAC assessment. Serum levels of vitamins A and E were determined in the hospital’s central laboratory with an ISO 15,189 accredited analysis method using reagents from Chromsystems (Gräfelfing, Germany) according to the manufacturer’s guidelines. Briefly, after protein precipitation, isocratic high-pressure liquid chromatography (HPLC) separation was carried out on an Agilent 1200 series system (Agilent Technologies, Santa Clara, CA, USA), and vitamins A and E were monitored by means of a diode array detector (Agilent) and quantified using calibrators by Chromsystems.

### 2.4. Immunohistochemical Staining of Liver Biopsies

First, 5 µm sections of 4% formaldehyde-fixed paraffin-embedded liver biopsies were cut, deparaffinized, and rehydrated. Antigen retrieval was performed in citrate buffer (pH = 6) in a microwave at 480 watts for 10 min. The endogenous peroxidase activity was blocked with 3% H2O2 for 10 min. The sections were permeabilized by 0.5% Triton X-100 (Carl Roth GmbH & Co. KG, Karlsruhe, Germany) in PBS Buffer (Life Technologies, Carlsbad, CA, USA) for 10 min. Unspecific bindings were blocked through 30 min of incubation in 10% immunized serum. The slides were then incubated with 10 µg/mL of the primary antibodies against human 4-HNE (ab46545, AbCam, Cambridge, UK) and human uncoupling protein 2 (UCP2; PA5-103176, Invitrogen, Carlsbad, CA, USA) in 1% BSA-PBS-0.5% Tween20 overnight at 4 °C. UCP2 and 4-HNE were chosen as tissue oxidative stress markers as they represent both mitochondrial ROS production as well as cell membrane lipid phospholipid peroxidation and have shown significant results in previous liver function experimental studies [37,38]. The bound primary antibodies were detected using species-specific VECTASTAIN ABC Elite Kits (Vector Laboratories, Inc., Newark, CA USA) and developed with DAB (DAKO, Glostrup, Denmark) as a chromogen. Sections were counterstained with hematoxylin. IHC staining was assessed by rating the extensity (% of positive cells) and intensity (0–3) on three different representative microscope fields. A semi-quantitative expression was then applied using the “quickscore” method by the multiplication of extensity with intensity (yielding values between 0 and 300) for each field [37].

### 2.5. Statistical Analysis

Data analysis and illustration were performed using SPSS statistics version 29 (IBM, Armonk, NY, USA) and Prism 10 (GraphPad Software; Boston, MA, USA). Categorial variables were displayed as the absolute number (n) and proportion (%) and continuous variables as the mean with standard deviation (SD) or median with interquartile range (IQR). An assessment of associations between the clinical parameters and immunohistochemical and laboratory markers was undertaken using Chi-Square/Fisher’s exact, Mann–Whitney U, Friedman, or Wilcoxon Signed Rank test as appropriate. A receiver operating characteristic (ROC) curve analysis was performed to determine the predictive values of several blood and tissue markers for PHLF, including the reporting of 95% confidence intervals (CIs). Ideal marker cutoffs were set by identifying where Youden’s J statistics were maximized on the ROC curve. For all analyses, *p*-values < 0.05 were considered statistically significant. This article is presented in accordance with the STROBE reporting checklist (STrengthening the Reporting of Observational studies in Epidemiology).

## 3. Results

### 3.1. Study Cohort

A total of 55 consecutive liver resection patients were enrolled in the study, of which 3 were excluded due to unresectability or a lack of samples. The characteristics and indications for surgery of the final cohort (n = 52) are described in Table 1. The median Charlson Comorbidity Index (CCI) and American Society of Anesthesiologists (ASA) classification was 6 and 2, respectively. Overall, 23.1% of liver specimens histologically showed steatosis or fibrosis, with no cases of underlying cirrhosis. The most common indications for resection included metastatic colorectal cancer (mCRC; 28.8%), cholangiocarcinoma (21.2%), and hepatocellular carcinoma (HCC; 21.2%). A large share of surgical procedures was classified as major liver resection (69.2%), and intraoperative inflow control (the Pringle maneuver) was used in 32.7% of cases (median: 23.5 min; IQR: 0; 51). The median time of surgery was 334 min, and most patients did not receive blood transfusions (67%). The rate of severe complications (Clavien–Dindo ≥3a) and death within 90 days postoperatively was 21.2% and 3.8%, respectively. In total, 13 patients (25%) experienced PHLF according to the ISGLS classification, of which 7 (13.8% of total) were classified as clinically relevant grade B or C.

### 3.2. Clinical Factors Associated with PHLF (Bivariate Analysis)

PHLF more commonly occurred after major resections compared to minor liver surgery (33.3% vs. 6.3%; *p* = 0.044). Also, patients undergoing surgery for cholangiocarcinoma compared to other indications experienced PHLF significantly more often (62% vs. 8%; *p* < 0.001). Of note, all patients with cholangiocarcinoma (n = 11) required a major hepatectomy compared to 25 of 41 patients with other indications (61.0%; *p* = 0.012). Patients developing PHLF had a median length of surgery of 8:19 h compared to 5:25 h in patients developing no PHLF (*p* = 0.008). The development of PHLF was a major contributor to overall postoperative complications: 90-day morbidity 100% vs. 38.5% (*p* < 0.001) and mortality 23% vs. 0% (*p* = 0.013). Consequently, patients with PHLF had a significantly prolonged overall median hospital stay (26 days vs. 9 days; *p* < 0.001).

### 3.3. Perioperative Oxidative Stress Blood Marker Dynamics after Hepatectomy

**Figure 1:**
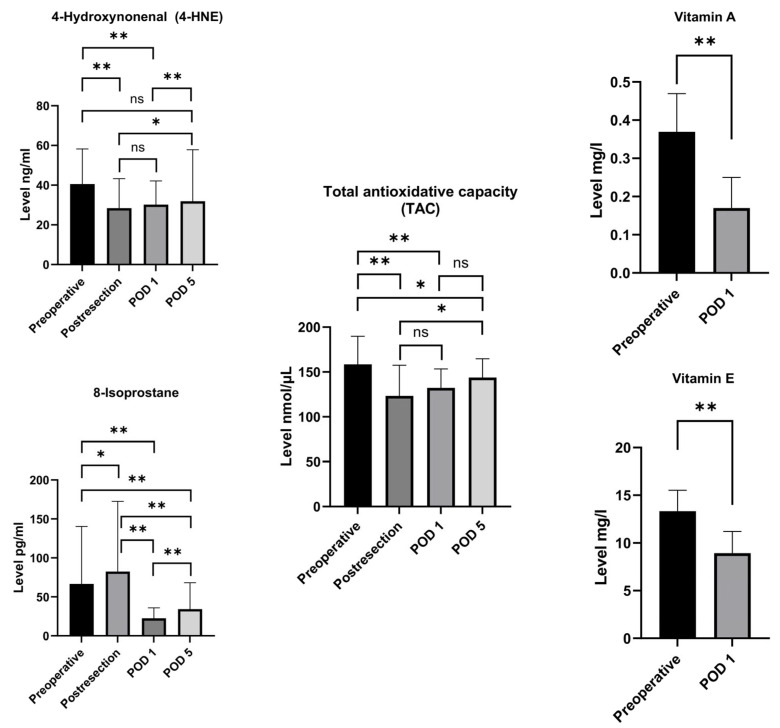
Dynamics of serum oxidative stress marker levels in the overall cohort (median with interquartile range/IQR). Abbreviations: POD1 and POD5 = postoperative days 1 and 5; * *p* < 0.05; ** *p* < 0.001; ns = not significant (Wilcoxon Signed Rank test for paired samples).

The analysis of oxidative stress marker dynamics (Figure 1) demonstrates a significant early postoperative increase in serum 8-isoprostane immediately post-resection from 66.8 pg/mL (IQR 33.9–140.5) to 82.7 pg/mL (IQR 37.7–172.8; *p* = 0.038), followed by a steep decline on POD1 (22.1 pg/mL; IQR 8.9–35.5; *p* < 0.001) and an increase on POD5 to 34.4 pg/mL (21.1–68.2; *p* < 0.001). Interestingly, 4-HNE had already decreased after completion of the resection phase from 40.6 ng/mL (IQR 20.9–58.2) to 28.5 ng/mL (IQR 14.5–43.2; *p* < 0.001), followed by a gradual increase on POD1 (30.3 ng/mL; IQR 18.1–42.1; *p* = 0.602) and POD5 (31.9 ng/mL; IQR 20.9–57.8; *p* < 0.001). Furthermore, the antioxidative markers significantly decreased perioperatively, potentially reflecting the consumption of antioxidative capacity. Preoperative levels of vitamin A compared to POD1 declined from 0.37 mg/L (IQR 0.24–0.47) to 0.17 mg/L (IQR 0.12–0.25; *p* < 0.001), vitamin E from 13.32 mg/L (IQR 10.93–15.53) to 8.92 mg/L (IQR 6.0–11.2; *p* < 0.001), and serum TAC from 158.5 nmol/µL (IQR 121.9–189.7) to 132.3 nmol/µL (IQR 88.5–157.5; *p* < 0.001).

### 3.4. Associations of Oxidative Stress Blood Markers with PHLF and PHLF-Related Risk Factors

**Figure 2:**
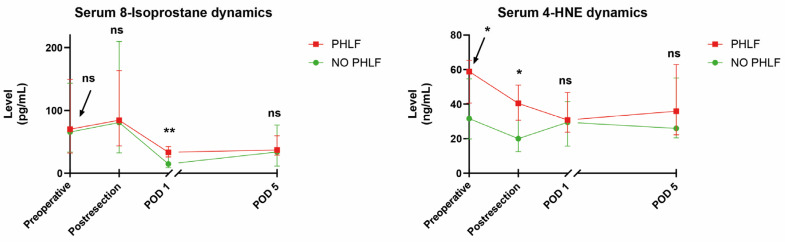
Dynamics of serum oxidative stress marker levels stratified by patients experiencing PHLF vs. no PHLF (medians with interquartile range/IQR). Abbreviations: 4-HNE = 4-hydroxynoneal; PHLF = post-hepatectomy liver failure; POD = postoperative day; * *p* < 0.05; ** *p* < 0.001; ns = not significant (Mann–Whitney U test).

Table 2 shows the median serum levels of 8-isoprostane, 4-HNE, vitamins A and E, and TAC according to different perioperative timepoints and stratified by the subsequent occurrence of PHLF. Overall, 8-isoprostane and 4-HNE were consistently higher in PHLF patients at all timepoints measured, and this was statistically significant for 8-isoprostane on POD1 and 4-HNE preoperatively and post-resection (Figure 2). Of note, no markers of antioxidative capacity were significantly different at any timepoint between the two groups of patients.

**Figure 3:**
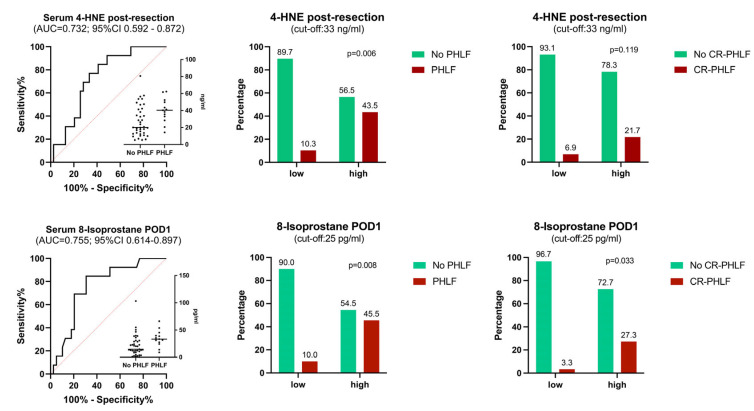
Predictive value (AUROC; **left**) of oxidative stress serum markers for development of PHLF after liver resection and event rates of PHLF (**middle**) and CR-PHLF (**right**) according to calculated cut-offs stratifying low- vs. high-risk patients (Chi-Square and Fisher’s exact test). 4-HNE = 4-hydroxynonenal; AUC = area under the curve; CI = confidence interval; CR-PHLF = clinically relevant post-hepatectomy liver failure (ISGLS grade B or C); PHLF = post-hepatectomy liver failure; POD = postoperative day.

An AUROC analysis of 8-isoprostane and 4-HNE at previously significant timepoints was performed to assess their value for PHLF prediction in the early perioperative phase. Preoperative 4-HNE showed an AUC of 0.703 (95% CI: 0.533–0.873; *p* = 0.030), post-resection 4-HNE of 0.732 (0.592–0.872; *p* = 0.013), and POD1 8-isoprostane of 0.755 (0.614–0.897; *p* = 0.006). As a next step, cut-offs were calculated for post-resection 4-HNE (33 ng/mL; sensitivity 76.9%, specificity 66.7%; NPV 89.7%) and POD1 8-isoprostane (25 pg/mL; sensitivity 76.9%, specificity 69.2%; NPV 90%). Figure 3 shows the AUROC of both markers and their rates of PHLF when applying these calculated cut-off values to the overall cohort.

Table 3 shows serum 8-isoprostane and 4-HNE levels stratified by clinical, surgical, and pathological variables potentially associated with PHLF development. The major factor for preoperatively increased 8-isoprostane levels was cholangiocarcinoma vs. all other entities combined (166 vs. 54.3 pg/mL; *p* = 0.014), which remained significant in the post-resection samples and on POD1. Interestingly, neither age over 60 years nor the presence of histological steatosis/fibrosis or a history of preoperative chemotherapy led to significantly increased 8-isoprostane or 4-HNE levels. However, 4-HNE was significantly increased in patients with steatosis/fibrosis post-resection (39.9 vs. 25.1 pg/mL; *p* = 0.039) and on POD1 (42.2. vs. 28.4 pg/mL; *p* = 0.008). The application of the Pringle maneuver was associated with a decreased median 8-isoprostane level on POD 1 (13.7 vs. 28.8 pg/mL; *p* = 0.015) and 4-HNE level post-resection (19.9 vs. 41.3 pg/mL; *p* = 0.031).

### 3.5. Immunohistochemical Analysis of Oxidative Stress

Table 4 lists the IHC staining intensity, extensity, and immunoreactivity scores for biopsies of both pre-resection (right and left) liver lobes, the FLR remaining in situ, and the resected liver specimen. When comparing these different specimens, there was a gradual increase in immunohistochemically detectable oxidative stress levels as measured by UCP2 and 4-HNE staining (overall, all *p* < 0.001). The mean/median UCP2 immunoscore of both preoperative biopsies combined was 133.3/150 (range 57.5–210) compared to 158.7/170 (range 70–270) in the FLR (*p* < 0.001) and 187.8/180 (range 90–285) in the resected specimen (*p* < 0.001). The mean/median 4-HNE immunoscore of both pre-resection biopsies combined was 93.6/80 (range 45–190) compared to 128.4/150 (range 60–190) in the liver remaining in situ (*p* < 0.001) and 161.3/170 (range 70–285) in the resected specimen (*p* < 0.001).

### 3.6. Associations of Liver Tissue Oxidative Stress with PHLF and PHLF-Related Risk Factors

**Figure 4:**
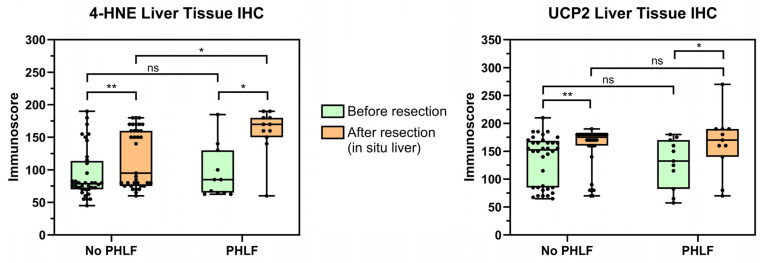
Liver tissue oxidative stress markers (4-HNE and UPC2) before and after resection (in situ liver) stratified by occurrence of PHLF. Boxplots: median with interquartile range (IQR) and min/max; dots: individual values. 4-HNE = 4-hydroxynonenal; PHLF = post-hepatectomy liver failure; UCP2 = uncoupling protein 2; * *p* < 0.05; ** *p* < 0.001; ns = not significant (Mann–Whitney U test).

There was a significant increase in median post-resection in situ liver 4-HNE and UCP2 immunoscores compared to pre-resection (Figure 4) independent of later development of PHLF (all *p* < 0.05). However, while the preoperative 4-HNE immunoscores between patients with and without subsequent PHLF were comparable (mean/median 93.1/78.8 vs. 92.2/85; *p* = 0.960), cases with PHLF presented significantly higher HNE levels in the post-resection in situ biopsies (mean/median: 159.1/170) compared to no PHLF (mean/median: 118.7/95; *p* = 0.015). This finding persisted when only patients with CR-PHLF (ISGLS PHLF B and C; n = 6) were compared to all others (no PHLF and ISGLS grade A PHLF), although not significantly (mean/median: 153.3/165 vs. 124.6/145; *p* = 0.158). When stained with UCP2, no significant difference in post-resection in situ biopsies between the PHLF and no-PHLF groups was observed (mean/median 153.6/170 vs. 157.1/175; *p* = 0.673). Of note, there was no significant difference in 4-HNE or UCP2 immunoscore levels in the resected liver specimens between patients with and without subsequent PHLF (4-HNE mean/median: 148.6/160 vs. 165.5/170; *p* = 0.390; UCP2 mean/median: 178.2/180 vs. 190.9/180; *p* = 0.468).

Table 5 shows pre- and post-resection UCP2 and 4-HNE immunoscores stratified by potential clinical, surgical, and pathological risk factors for PHLF (age > 60 years, steatosis/fibrosis, cholangiocarcinoma, neoadjuvant chemotherapy, the Pringle maneuver, intraoperative transfusion, and extent of resection). There was no significant association of preoperative UCP2 or 4-HNE levels with any of the above-mentioned risk factors. However, in post-resection in situ livers, both UCP2 and 4-HNE immunoscore levels were significantly elevated in cholangiocarcinoma patients compared to all other indications combined (190.0 vs. 151.9; *p* = 0.021 and 163.3 vs. 119.9; *p* = 0.005). This was further underlined by the finding of higher postoperative 4-HNE immunoscore levels in patients without vs. with preoperative chemotherapy (134.5 vs. 98.6; *p* = 0.012), most likely due to cholangiocarcinoma patients not receiving neoadjuvant treatment in this cohort.

### 3.7. Intraoperative PHLF Prediction Combining Post-Resection Tissue and Serum Markers

**Figure 5:**
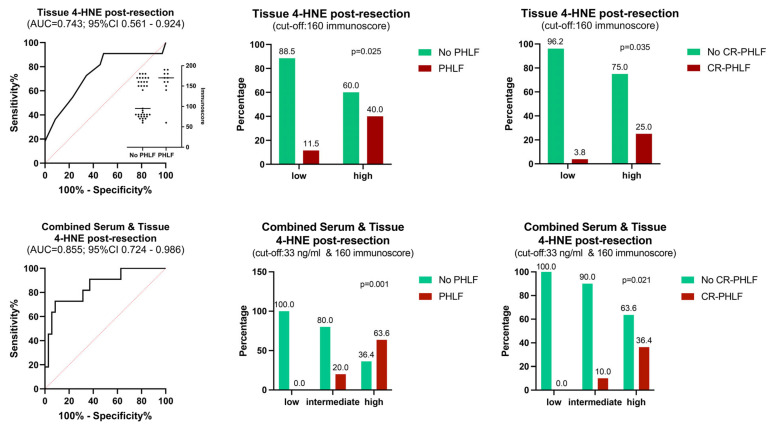
Predictive value of tissue 4-HNE alone (**top** panel) or in combination with serum 4-HNE (**bottom** panel) for PHLF after liver resection (**left**). Event rates of PHLF (**middle**) and CR-PHLF (**right**) according to low- (vs. intermediate) vs. high-risk cut-offs (Chi-Square and Fisher’s exact test). 4-HNE = 4-hydroxynonenal; AUC = area under the curve; CI = confidence interval; CR-PHLF = clinically relevant post-hepatectomy liver failure (ISGLS Grade B or C); PHLF = post-hepatectomy liver failure; POD = postoperative day.

**Figure 6:**
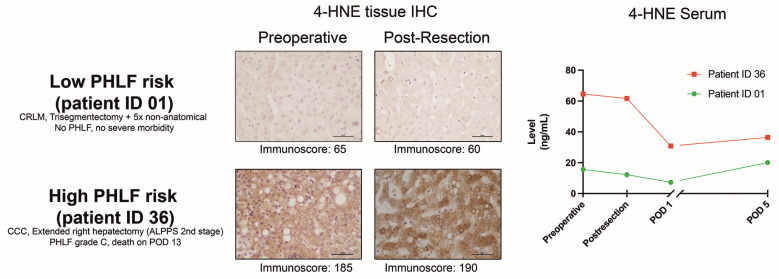
Patient examples with low and high PHLF risk according to combined 4-HNE post-resection in situ biopsy IHC and serum level cut-offs (scale bar on IHC images = 50 µm). 4-HNE = 4-hydroxynonenal; ALPPS = associating liver partition and portal vein ligation for staged hepatectomy; CCC = cholangiocarcinoma; CRLM = colorectal cancer liver metastases; IHC = immunohistochemistry; PHLF = post-hepatectomy liver failure; POD = postoperative day.

An AUROC analysis was performed to assess the value of 4-HNE tissue IHC for PHLF prediction in the immediate post-resection phase. The AUC for 4-HNE immunoscores (Figure 5) from biopsies taken from the in situ FLR was 0.743 (0.561–0.924; *p* = 0.016), and Youden’s index cut-off calculation resulted in an immunoscore value of 160 (sensitivity 72.7%; specificity 66.7%; NPV 88.5%). To further improve the predictive value, we exemplarily combined the IHC levels with the post-resection 4-HNE serum levels (AUC 0.732) through binary logistic regression (Figure 5). This approach of a tissue and serum biopsy taken at the end of the liver resection resulted in an AUC to predict PHLF of 0.855 (95% CI 0.724–0.986; *p* < 0.001) and CR-PHLF of 0.767 (95% CI 0.555–0.979; *p* = 0.037). While patients with low serum (cut-off of 33 pg/mL) and tissue 4-HNE (cut-off immunoscore of 160) values had a 0% PHLF and 0% CR-PHLF rate, this gradually increased to 20% PHLF and 10% CR-PHLF when one factor was above the cut-off, and 63.6% PHLF and 36.4% CR-PHLF with both factors high (*p* = 0.001 and *p* = 0.021, respectively). This was also associated with high 90-day mortality (18.2%) compared to patients with none or one marker above the cut-off (0% mortality; *p* = 0.036). Figure 6 exemplarily depicts 4-HNE histology IHC slides and serum dynamics for a low- and high-risk patient according to this proposed risk stratification principle.

### 3.8. Potential Influence of Malignant Entities and Tumor Burden on Oxidative Stress Levels

To explore the possibility of pronounced oxidative stress levels in cholangiocarcinoma patients and a potential link to tumor burden, we performed subgroup analyses comparing cholangiocarcinoma (CCC), hepatocellular carcinoma (HCC), and CRLM patients only (n = 37; Supplementary Data). Since direct comparison between these entities by TNM stage would not be permissible due to different staging systems in primary and secondary liver malignancies, we first assessed maximum tumor size as a surrogate marker for tumor burden (Supplementary Data A). HCC patients numerically had the largest median tumor size (80 mm) compared to CRLM and CCC (50 mm each) but without statistical significance (*p* = 0.358). Median preoperative and pre-resection serum and local liver tissue oxidative stress markers were consistently highest in CCC, although this reached only borderline statistical significance for 8-isoprostane: median 166.6 pg/mL in CCC compared to 54.3 pg/mL in CRLM and 79.2 pg/mL in HCC (*p* = 0.058). Overall, there was no (positive) correlation between maximum tumor size and preoperative serum 8-isoprostane levels (rho: −0.252; *p* = 0.134).

Next, CCC and HCC patient groups (n = 11 each) were explored individually by tumor stage. In CCC patients, T2 versus T1 stage and nodal positivity versus nodal negativity were associated with increasing serum and tissue marker oxidative stress levels (Supplementary Data B). Despite the limited sample size and statistical power, this was significant for preoperative serum 4-HNE when stratified by T stage with 29.5 ng/mL (T1) compared to 64.6 ng/mL (T2; *p* = 0.013).

In contrast, there was no clear association of T stage with increasing oxidative stress levels in HCC (Supplementary Data C). A potential link with pathological N status could not be explored due to the limited sample size (N0: n = 7, N1: n = 1, and Nx: n = 3).

## 4. Discussion

As previously described in a systematic review by our group, the creation of reactive oxygen species and the resulting oxidative stress in the field of hepatic surgery have so far mainly been addressed in animal models [5]. To the best of our knowledge, the present work represents the first human prospective study to evaluate serum and immunohistochemical markers for oxidative stress in regards to its correlation with hepatic function and potential to predict PHLF according to established criteria (the ISGLS) [23]. Previous evaluations in clinical settings were most commonly derived from liver transplantation rather than liver resection patients [5,14,19,38,39]. Representing one of the few human liver resection studies on oxidative stress, a previous work by Schwarz et al. utilized static oxidation-reduction potential markers (sORPs) and antioxidant capacity (cORP) in the perioperative period through the application of the RedoxSYS^®^ diagnostic system [13]. They mainly focused on the (significant) association of oxidative stress and inflammation with overall outcome (severe complications) and did not specifically assess postoperative hepatic function. Similarly, another prospective observational study recently published by a British group evaluated perioperative redox changes in patients undergoing hepato-pancreato-biliary cancer surgery [14]. Their cohort was inhomogeneous as they included not only liver resections (55%) but also major pancreatic resections (32%) and even palliative surgery (13%). They mainly describe the redox serum marker dynamics from baseline to the end of surgery and POD1, showing that some of these markers at baseline are associated with overall postoperative morbidity. No subgroup analysis of solely liver resection patients or a specific assessment of PHLF was reported.

In contrast, the present pilot study utilized a rather homogeneous cohort of consecutive patients undergoing open elective liver surgery only. Although no cases with cirrhotic livers were recruited (23% steatosis or fibrosis and 15% preoperative chemotherapy), a major hepatectomy rate (>3 segments) of 69% and a resulting PHLF rate of 25% (14% CR-PHLF) provided adequate statistical power to assess the endpoints of interest. Expectedly, PHLF mainly occurred after major resections and in cholangiocarcinoma patients and was linked to longer surgeries and hospital stays and more overall complications.

The analysis of oxidative stress serum markers in this study shows several relevant findings, with 8-isoprostane and 4-HNE demonstrating distinct perioperative dynamics. While 8-isoprostane still increased post-resection compared to preoperative levels and was not statistically significantly different on both timepoints when stratifying patients by the occurrence of PHLF, this marker signalized utility for PHLF prediction on POD1. In contrast, median 4-HNE was already significantly different preoperatively between the two groups and decreased at the end of the parenchymal transection, maintaining its predictive potential. Antioxidant markers, including vitamins A and E, as well as total antioxidative capacity (TAC), showed uniform patterns of consumption early postoperatively. Surprisingly, age > 60, presence of steatosis or fibrosis, and preoperative chemotherapy did not significantly increase the preoperative serum levels of oxidative stress markers, and this was also confirmed on a tissue level. This finding is in contrast to previous experimental studies suggesting an increase in serum oxidative stress markers in fibrotic liver parenchyma changes and a significant role of lipid peroxidation in alcoholic liver disease [34,40,41]. However, in our study, serum 4-HNE was significantly higher post-resection and on POD1 in patients with steatotic or fibrotic liver parenchyma. This is in line with a previous experimental study, which demonstrated that mice with diabetic steatotic livers presented distinct oxidative stress generation peaking at 30 min and lasting for at least 8 h after a two-third hepatectomy compared to lean controls [42].

As a finding of particular interest, our study suggests that cholangiocarcinoma seems profoundly associated with consistently increased perioperative oxidative stress measured on a systemic serum and local non-tumoral hepatic tissue level. Previous results supporting these observations have been reported by a Japanese group in an experimental human and animal model setting [16]. They showed that serum levels of reactive oxygen metabolites were significantly higher in cholangiocarcinoma patients with poor outcomes, while there was no difference in pancreatic cancer patients. In their diabetic mouse-based cholangiocarcinoma model, an add-on antioxidant therapy to chemotherapy improved the dysregulated oxidative stress balance. To what extent the increased oxidative stress levels in cholangiocarcinoma patients are caused by the tumor environment itself or the associated liver parenchyma damage (cholestasis, cholangitis, or fibrosis) remains debatable. As a side note, in our study, all patients had preoperative bilirubin levels within the normal range, and there was no significant correlation between bilirubin and any perioperative oxidative stress markers (these results are not reported in detail). However, subgroup analyses provided in the Supplementary Data suggest that specifically in cholangiocarcinoma patients, there could be a pattern of increasing a priori systemic and local hepatic oxidative stress levels in higher tumor stages and nodal-positive tumors. The differences in early post-resection and POD1 8-isoprostane serum levels between cholangiocarcinoma and non-cholangiocarcinoma patients in our study could have been further influenced by a need for more extensive liver resections in this specific indication (100% vs. 61% major resection; *p* = 0.012). Since even comparably small tumors in perihilar cholangiocarcinoma (Klatskin tumors) often require substantial hepatectomies depending on vascular and bile duct involvement, the tumor size/tumor burden alone does not constitute a reliable parameter for comparison to other tumor types, as underlined by the correlation analysis between tumor size and preoperative serum 8-isoprostane (Supplementary Data A). To further elucidate these interesting findings in future studies, an evaluation of perioperative oxidate stress levels in a larger cohort comparing both intrahepatic and perihilar cholangiocarcinoma patients seems worthwhile.

The application of unselective inflow occlusion with a Pringle maneuver interestingly decreased 8-isoprostane levels on POD 1 and 4-HNE levels post-resection, potentially through ischemic preconditioning and diminished intraoperative stress with less acute bleeding, although this hypothesis could not be further evaluated with the available data. Comparable findings have been demonstrated in a recent randomized controlled trial applying direct (portal vein clamping) or remote (upper limb tourniquet) ischemic preconditioning in patients undergoing partial hepatectomy [43]. The study showed that both techniques, indeed, result in highly significant increased (antioxidant) superoxide dismutase (SOD) and lower TNF-α serum levels compared to patients without intervention.

Focusing on intra-tissue oxidative stress levels, measurement through IHC revealed reliable and reproducible results, with consistent increments of 4-HNE and UCP2 immunoscores from pre-resection to post-resection FLR and resected specimens (all *p* < 0.001). For early prediction of PHLF, specifically post-resection tissue 4-HNE could represent a valuable marker (AUC = 0.743, p = 0.016). Its predictive potential is further increased when combined with the simultaneous assessment of serum 4-HNE levels (combined AUC = 0.855, *p* < 0.001). In a clinical setting, the measurement of serum and IHC intra-tissue 4-HNE levels at the end of the resection phase could help in the early identification of patients at high risk for developing PHLF within the first days after surgery. Patients with both factors over the calculated cut-offs (33 pg/mL and an immunoscore of 160) had a striking rate of 63.6% PHLF (36.4% CR-PHLF) compared to 0% PHLF (0% CR-PHLF) when both factors were below the cut-offs (*p* = 0.001 and *p* = 0.021). This was also associated with a high 90-day mortality (18.2% vs. 0%; *p* = 0.036). These findings contribute to the previously described perioperative markers applicable for PHLF prediction, such as lactate and the von Willebrand Factor Antigen [22,24,26,44,45]. Certainly, the present results require external validation in a larger multicenter cohort. Ultimately, these markers could be implemented in machine learning algorithms to aid clinicians in decision-making regarding perioperative risk stratification, intensity of observation, early diagnostic interventions, and prophylactic therapies [46].

The limitations of this pilot study include a moderate sample size and the lack of an independent, external validation cohort, which precluded further multivariable and detailed subgroup analysis. To ensure the high quality and validity of our results, a strict prospective protocol was applied, including all consecutive elective liver resections at our center over the study period. Importantly, the outcome parameters were cross-checked with our auditable, quality-controlled clinical registry database [47]. This study, first and foremost, focuses on reporting the principal dynamics of perioperative oxidative and antioxidative markers during liver resection. As such, their association with PHLF and predictive potential is mainly descriptive and does not necessarily prove causality or allow for a conclusion that any intervention on oxidative stress levels could ultimately lead to improved postoperative outcomes, especially in terms of liver function. Therefore, future research should focus on assessing and validating a causal association between increased oxidative stress and PHLF in large-scale cohorts and be sufficiently powered to further evaluate the possible influence of the type of malignancy, tumor burden, and underlying liver disease on systemic oxidative stress levels. Subsequently, therapeutic approaches could be evaluated in patients with specifically high ROS levels and risk for PHLF, such as cholangiocarcinoma or HCC patients with fibrotic/steatotic livers planned for major hepatectomy, as suggested by the results of present and previous publications [12,16,18,36,48].

## 5. Conclusions

This study reveals distinct patterns of perioperative oxidative stress levels in patients undergoing elective liver resections. A combination of serum and liver tissue markers significantly predicts postoperative liver dysfunction as defined by the ISGLS. Patients with cholangiocarcinoma seem particularly associated with consistently increased perioperative oxidative stress, and this was more pronounced in cases with higher tumor stages. The latter cohort represents a potential target cohort of interest for future exploratory and interventional therapeutic studies.

## Figures and Tables

**Table 1:** Patient characteristics and surgical and perioperative details (n = 52).

Age (years), median (IQR)	58.5 (51; 68.8)
Male sex	31 (59.6%)
BMI, median (IQR)	25.5 (16.2 to 40.3)
Charlson Comorbidity Index (CCI), median (IQR)	6 (3; 7)
ASA Classification	
1	5 (9.6%)
2	38 (73.1%)
3	9 (17.3%)
Liver parenchymal disease	
Steatosis	8 (15.4%)
Fibrosis	4 (7.7%)
Cirrhosis	0 (0%)
Preoperative chemotherapy	8 (15.4%)
Indication for surgery	
CRLM	15 (28.8%)
CCC	11 (21.2%)
HCC	11 (21.2%)
Benign/pre-malignant lesions	6 (11.5%)
Non-CRLM	5 (9.6%)
Other primary liver tumors	2 (3.8%)
Echinococcus	1 (1.9%)
Chronic cholangitis	1 (1.9%)
**Surgical procedures and perioperative outcomes**	
Major resection (>3 segments)	36 (69.2%)
Liver resection type	
Right hemihepatectomy	13 (25%)
Left hemihepatectomy	3 (5.8%)
Extended right hepatectomy	10 (19.2%)
Extended left hepatectomy	6 (11.5%)
Bisegmentectomy	7 (13.5%)
Other anatomical and non-anatomical resections	13 (25%)
Inflow occlusion/Pringle maneuver applied	17 (32.7%)
Operative time (minutes), median (IQR)	334 (275; 433)
Intraoperative blood loss (mL), median (IQR)	700 (413; 1000)
Intraoperative transfusion (number of packs)	
1	6 (12%)
>1	11 (21%)
Length of hospital stay (days), median (IQR)	10 (8; 19)
90-day morbidity overall	28 (53.8%)
Severe complications (Clavien–Dindo >3a)	11 (21.2%)
90-day mortality	2 (3.8%)
PHLF (ISGLS); clinically relevant PHLF (ISGLS Grade B/C)	13 (25%); 7 (13.5%)

All data are given as absolute numbers (%) or medians with interquartile range (IQR). Abbreviations: ASA = American Society of Anesthesiologists; BMI = body mass index; CCC = cholangiocarcinoma; CRLM = colorectal cancer liver metastases; HCC = hepatocellular carcinoma; ISGLS = International Study Group for Liver Surgery; PHLF = post-hepatectomy liver failure.

**Table 2:** Oxidative stress blood markers stratified by occurrence of PHLF (n = 13) vs. no PHLF (n = 39).

	Preoperatively	Post-Resection	POD 1	POD 5
**8-Isoprostane (pg/mL)**				
No PHLF	65.5 (34.0–143.2)	80.8 (32.3–209.8)	14.7 (8.9–30.7)	33.9 (11.4–76.8)
PHLF	70.2 (32.1–149.6)	84.5 (43.6–163.5)	33.3 (25.9–42.5)	37.2 (28.6–59.7)
*p*-value *	0.792	0.983	**0.006**	0.691
**4-HNE (ng/mL)**				
No PHLF	31.6 (19.8–54.6)	20.0 (12.5–41.04)	29.5 (15.7–41.5)	26.0 (20.5–55.1)
PHLF	58.9 (40.6–65.4)	40.4 (30.7–51.1)	30.8 (23.8–46.8)	35.9 (22.3–62.9)
*p*-value *	**0.029**	**0.013**	0.296	0.820
**Vitamin A (mg/L)**				
No PHLF	0.37 (0.23–0.47)	n/a	0.2 (0.14–0.28)	n/a
PHLF	0.34 (0.25–0.44)	n/a	0.13 (0.1–0.24)	n/a
*p*-value *	0.848		0.097	
**Vitamin E (mg/L)**				
No PHLF	13.32 (10.89–15.51)	n/a	9.31 (7.42–11.41)	n/a
PHLF	11.32 (10.31–15.12)	n/a	7.2 (6.02–9.51)	n/a
*p*-value *	0.719		0.170	
**TAC (nmol/μL)**				
No PHLF	154.5 (117.9–188.9)	123.3 (88.5–146.8)	120.3 (103.1–152.8)	143.7 (116.5–166.6)
PHLF	166.3 (137.6–214.8)	156.3 (101.8–163.5)	148.4 (98.1–162.4)	145.2 (110.0–161.9)
*p*-value *	0.139	0.148	0.410	1.000

* Mann–Whitney U test; all data are given as medians with interquartile range. Abbreviations: n/a = not assessed; PHLF = post-hepatectomy liver failure; POD = postoperative day; TAC = total antioxidative capacity; 4-HNE = 4-hydroxynonenal.

**Table 3:** Serum 8-isoprostane and 4-HNE levels stratified by clinical, surgical, and pathological variables.

	8-Isoprostane (pg/mL)			4-HNE (ng/mL)		
	Preoperative	Post-Resection	POD 1	Preoperative	Post-Resection	POD 1
**Age > 60 years**						
No	59.9 (19.4–105.7)	68.7 (30.7–172.8)	17.5 (11.0–31.6)	45.5 (28.0–61.3)	30.6 (17.1–46.1)	30.3 (17.0–45.0)
Yes	73.9 (45.4–146.6)	82.7 (49.6–190.5)	23.6 (12.3–39.0)	31.0 (18.8–52.2)	24.5 (13.7–41.4)	29.9 (18.3–42.1)
*p*-value *	0.212	0.497	0.388	0.069	0.582	0.890
**Steatosis/Fibrosis**						
No	64.1 (33.9–147.6)	84.8 (37.7–172.8)	21.7 (11.0–35.1)	39.4 (19.7–58.0)	25.1 (12.8–41.2)	28.4 (15.2–39.0)
Yes	69.1 (22.6–117.7)	69.7 (30.6–176.2)	27.6 (15.3–38.0)	42.5 (25.4–62.5)	39.9 (20.1–54.8)	42.2 (29.7–56.7)
*p*-value *	0.680	0.704	0.799	0.441	**0.039**	**0.008**
**Cholangiocarcinoma**						
No	54.3 (30.6–103.3)	58.6 (31.4–163.2)	14.7 (8.9–33.5)	38.8 (21.7–55.3)	23.8 (14.5–40.9)	30.2 (16.5–44.6)
Yes	166.6 (46.7–229.2)	163.6 (80.7–233.0)	31.7 (24.3–39.7)	54.6 (19.8–64.6)	41.3 (14.2–49.8)	30.3 (20.8–31.0)
*p*-value *	**0.014**	**0.043**	**0.007**	0.330	0.161	0.823
**Neoadjuvant CTX**						
No	66.8 (34.4–140.5)	84.8 (42.4–195.9)	23.5 (12.1–38.8)	42.5 (23.6–60.6)	32.9 (18.0–46.1)	30.4 (21.9–43.4)
Yes	64.0 (19.4–140.0)	38.25 (24.2–143.5)	13.4 (9.2–27.1)	23.8 (16.4–48.9)	11.5 (6.2–25.7)	18.5 (8.1–33.9)
*p*-value *	0.718	0.127	0.140	0.108	**0.024**	0.065
**Pringle maneuver**						
No	77.6 (48.1–157.0)	50.6 (27.5–167.2)	28.8 (20.2–39.4)	42.9 (25.5–58.2)	41.3 (20.6–51.1)	30.3 (19.0–46.8)
Yes	54.3 (31.3–117.9)	84.5 (38.0–174.3)	13.7 (8.4–35.5)	38.8 (19.6–58.9)	19.9 (13.5–38.4)	29.1 (15.7–39.5)
*p*-value *	0.230	0.592	**0.015**	0.501	**0.031**	0.532
**Intraop. Transfusion**						
No	77.6 (33.7–143.2)	84.5 (30.5–174.3)	22.2 (11.5–36.2)	41.2 (19.8–55.4)	20.5 (14.2–42.3)	29.5 (17.2–40.7)
Yes	49.4 (34.8–122.7)	80.7 (43.6–185.8)	27.5 (10.2–37.1)	39.1 (28.4–61.8)	35.6 (16.5–45.5)	30.5 (19.1–46.5)
*p*-value *	0.552	0.891	0.992	0.565	0.396	0.704
**Major/Minor**						
Minor	59.9 (37.3–128.8)	66.6 (25.0–151.1)	24.8 (11.9–39.5)	33.7 (20.0–55.2)	29.9 (18.3–50.4)	24.8 (11.9–39.5)
Major	69.2 (31.9–145.3)	84.8 (38.2–205.7)	22.5 (11.1–32.9)	41.7 (22.4–61.2)	30.3 (17.0–40.4)	22.5 (11.1–32.9)
*p*-value *	0.633	0.326	0.586	0.341	0.874	0.552

* Mann–Whitney U test; all values are given as medians with interquartile range. Abbreviations: POD = postoperative day; CCC = cholangiocellular carcinoma; CTX = chemotherapy; intraop. = intraoperative.

**Table 4:** Immunohistochemical analysis of oxidative stress markers according to different biopsy timepoints.

	Pre-Resection		Post-Resection		*p* *
	Right Liver Lobe Mean (SD); Median	Left Liver Lobe Mean (SD); Median	In situ FLR Mean (SD); Median	Resected Liver Mean (SD); Median	
UCP2—Intensity	1.66 (± 0.48); 2	1.63 (± 0.53); 2	1.84 (±0.42); 2	2.13 (± 0.41); 2	<0.001
UCP2—Extensity	78.1 (± 8.9); 80	81.6 (± 9.6); 80	85.2 (± 7.2); 90	87.8 (± 8.0); 90	<0.001
UCP2—Score	131.7 (± 45.4); 150	134.0 (± 48.3); 150	158.7 (± 42.3); 170	187.8 (± 42.6); 180	<0.001
4-HNE—Intensity	1.23 (± 0.42); 1	1.28 (± 0.46); 1	1.59 (± 0.50); 2	1.91 (± 0.56); 2	<0.001
4-HNE—Extensity	71.5 (± 12.6); 72.5	74.8 (± 12.0); 77.5	79.9 (± 8.5); 80	84.1 (± 8.2); 85	<0.001
4-HNE—Score	89.3 (± 39.9); 75	97.3 (± 42.1); 80	128.4 (± 46.0); 150	161.3 (± 52.9); 170	<0.001

* Friedman test comparing combined right and left pre-resection values vs. in situ and resected liver. Abbreviations: 4-HNE = 4-hydroxynonenal; FLR = future liver remnant; Score = immunoscore; SD = standard deviation; UCP2 = uncoupling protein 2.

**Table 5:** Tissue oxidative stress (IHC scores) stratified by clinical, surgical, and pathological variables.

	Pre-Res. UCP2	Post-Res. UCP2	Pre-Res. 4-HNE	Post-Res. 4-HNE
**Age > 60 years**				
No	134.2 (42.5)	157.8 (39.7)	85.9 (30.8)	131.5 (47.3)
Yes	132.4 (46.5)	160.0 (45.8)	101.6 (45.0)	125 (45.4)
*p*-value *	0.957	0.648	0.579	0.808
**Steatosis/fibrosis**				
No	131.8 (46.6)	154.9 (41.9)	88.6 (34.6)	123.8 (47.3)
Yes	137.7 (36.8)	169.2 (43.4)	108.1 (47.7)	141.3 (40.7)
*p*-value *	0.705	0.884	0.566	0.352
**Cholangiocarcinoma**				
No	131.0 (44.1)	151.9 (41.2)	90.5 (35.4)	119.9 (45.3)
Yes	143.1 (44.9)	190.0 (33.8)	106.7 (51.3)	163.3 (30.8)
*p*-value *	0.357	**0.021**	0.409	**0.005**
**Neoadjuvant CTX**				
No	135.0 (43.0)	161.1 (40.6)	97.9 (40.2)	134.5 (44.1)
Yes	123.6 (52.0)	145.7 (52.2)	68.9 (14.4)	98.6 (43.2)
*p*-value *	0.549	0.616	0.120	**0.012**
**Pringle maneuver**				
No	134.8 (41.1)	159.2 (51.3)	110.4 (49.2)	146.9 (33.7)
Yes	132.7 (45.8)	158.5 (39.5)	86.5 (31.7)	121.1 (48.5)
*p*-value *	0.843	0.354	0.305	0.297
**Intraop. transfusion**				
No	129.8 (44.2)	162.3 (41.6)	93.4 (39.7)	131.0 (45.3)
Yes	140.0 (44.3)	151.3 (44.2)	94.1 (38.3)	123.4 (48.4)
*p*-value *	0.444	0.351	0.973	0.437
**Major/minor**				
Minor	132.7 (46.2)	160.7 (37.3)	108.3 (33.5)	123.2 (44.3)
Major	133.6 (43.7)	157.7 (44.9)	86.7 (46.0)	130.6 (47.2)
*p*-value *	0.991	0.859	0.140	0.457

All data are given as means (standard deviation); * Mann–Whitney U test; pre-resection IHC scores are mean of bilobar biopsies. Abbreviations: 4-HNE = 4-hydroxynonenal; bx = biopsies; CTX = chemotherapy; IHC = immunohistochemistry; intraop. = intraoperative; UCP2 = uncoupling protein 2.

## Data Availability

The raw data supporting the conclusions of this article will be made available by the authors on request.

## Supplementary Materials

The following supporting information can be downloaded at: https://www.mdpi.com/article/10.3390/antiox13050590/s1.
