# Supplementary material for: The Interplay between Perioperative Oxidative Stress and Hepatic Dysfunction after Human Liver Resection: A Prospective Observational Pilot Study
Source: Antioxidants (Basel). 2024 May 11;13(5):590. doi: 10.3390/antiox13050590 (PMC11118143; doi:10.3390/antiox13050590)

Supplementary data

The interplay between perioperative oxidative stress and hepatic dysfunction after human liver resection: a prospective observational pilot study

Primavesi F et al., Antioxidants, 2024

A) Comparison of colorectal cancer liver metastases, cholangiocarcinoma and hepatocellular carcinoma patients (n=37)

	CRLM (n=15)	CCC (n=11)	HCC (n=11)	*P* *
Maximum tumour size (mm)	50 (30; 65)	50 (27; 62)	80 (40; 100)	0.358
Preoperative serum 8-isoprostane (pg/ml)	54.3 (33.7; 85.6)	166.6 (46.7; 229.2)	79.2 (34.7; 129.3)	0.058
Preoperative serum 4-HNE (ng/ml)	23.5 (18.5; 45.5)	54.6 (19.8; 64.6)	42.9 (23.2; 56.2)	0.264
Pre-resection UCP-2 immunoscore	145(70;165)	160 (98.75; 180)	132.5 (85; 175)	0.463
Pre-resection 4-HNE immunoscore	80 (67.5; 127.5)	85 (65; 167.5)	72.5 (70; 120)	0.752

All values are median with IQR; * = p-values calculated with Kruskal-Wallis test;
4-HNE = 4-hydroxynonenal; CCC = cholangiocarcinoma; CRLM = colorectal liver metastases; HCC = hepatocellular carcinoma; UCP-2 = uncoupling protein 2.

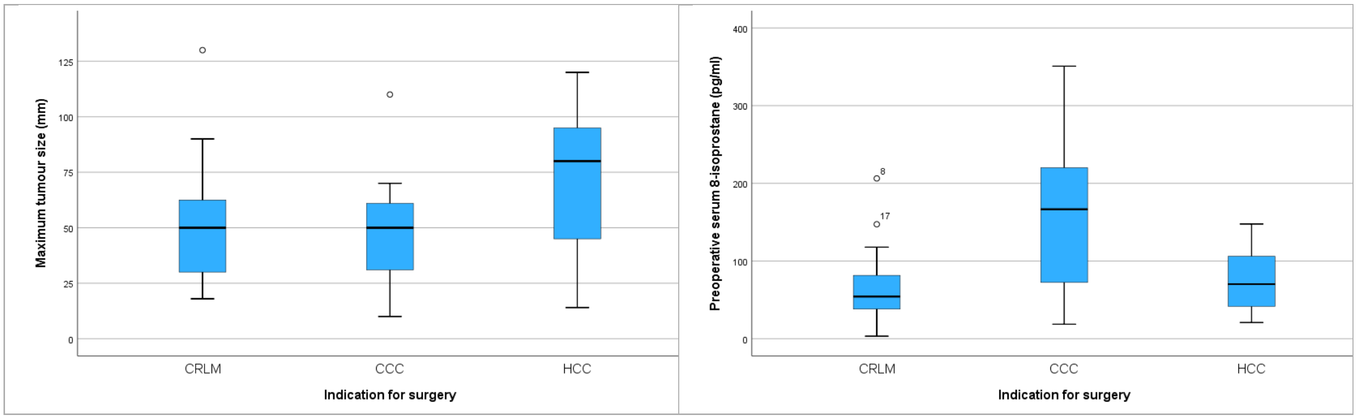

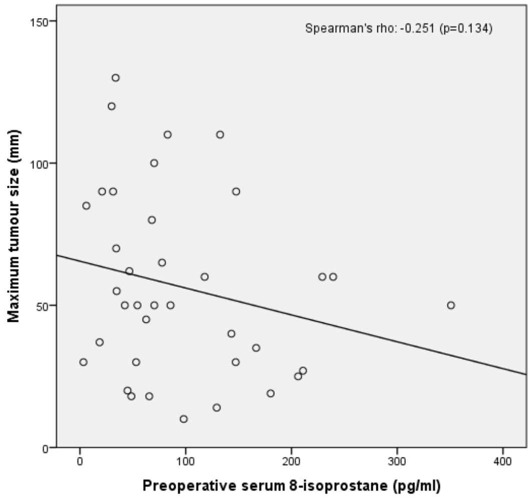

B) Subgroup analysis of cholangiocarcinoma patients only (n=11)

	T1 (n=6)	T2 (n=5)	*P**
Preoperative serum 8-isoprostane (pg/ml)	149.6 (39.7; 195.1)	210.9 (66.2; 290)	0.465
Preoperative serum 4-HNE (ng/ml)	29.5 (17.2; 46.9)	64.6 (57.9; 66.4)	0.013
Pre-resection UCP-2 immunoscore	132.5 (90.6; 176.3)	170 (112.5; 180)	0.539
Pre-resection 4-HNE immunoscore	71.3 (65; 154.4)	85 (73.8; 170)	0.459
	N- (n=8)	N+ (n=3)	*P**
Preoperative serum 8-isoprostane (pg/ml)	115.3 (37.4; 224.6)	210.9 (166.6; n/c)	0.307
Preoperative serum 4-HNE (ng/ml)	40.6 (17.9; 63.8)	61.2 (54.6; n/c)	0.183
Pre-resection UCP-2 immunoscore	150 (82.5; 175)	172.5 (160; n/c)	0.303
Pre-resection 4-HNE immunoscore	77.5 (65; 180)	120 (85; n/c)	0.460

All values are median with IQR; * = p-values calculated with Mann-Whitney-U test;
4-HNE = 4-hydroxynonenal; n/c = not computable; UCP-2 = uncoupling protein 2.

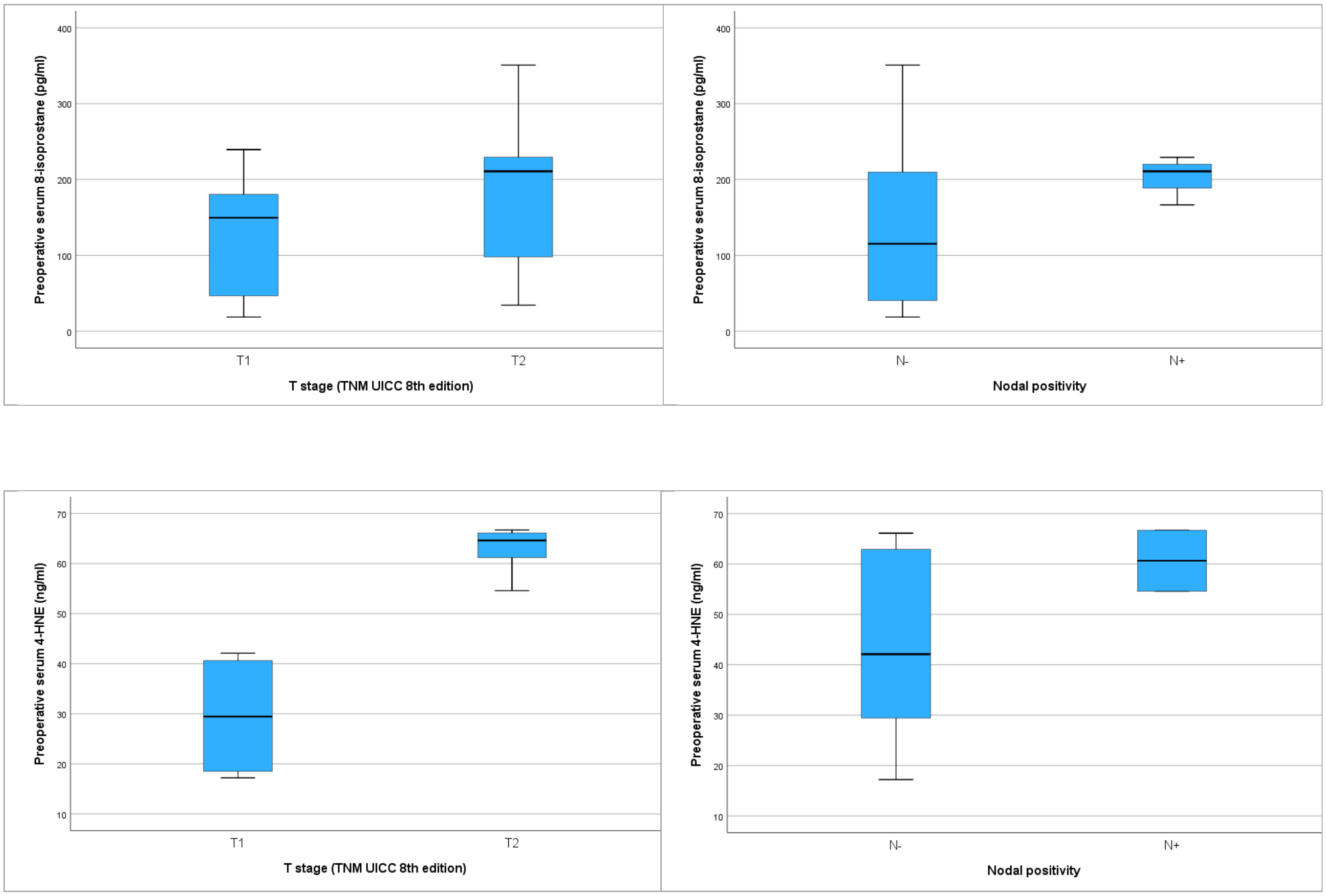

C) Subgroup analysis of hepatocellular carcinoma patients only (n=11)

	T1 (n=5)	T2 (n=2)	T3 (n=2)	T4 (n=2)	*P**
Preoperative serum 8-isoprostane (pg/ml)	82.9 (52.5; 145.5)	59.5 (48.6; n/c)	25.4 (20.9; n/c)	98.7 (68; n/c)	0.167
Preoperative serum 4-HNE (ng/ml)	42.9 (26.8; 49.7)	37.5 (31.6; n/c)	51.6 (14.5; n/c)	63.5 (15.7; n/c)	0.985
Pre-resection UCP-2 immunoscore	150 (110; 180)	120 (85; n/c)	133.8 (57.5; n/c)	83.8 (82.5; n/c)	0.418
Pre-resection 4-HNE immunoscore	120 (72.5; 142.5)	72.5 (70; n/c)	73.8 (67.5; n/c)	67.5 (62.5; n/c)	0.274

All values are median with IQR; * = p-values calculated with Kruskal-Wallist test;
4-HNE = 4-hydroxynonenal; n/c = not computable; UCP-2 = uncoupling protein 2.

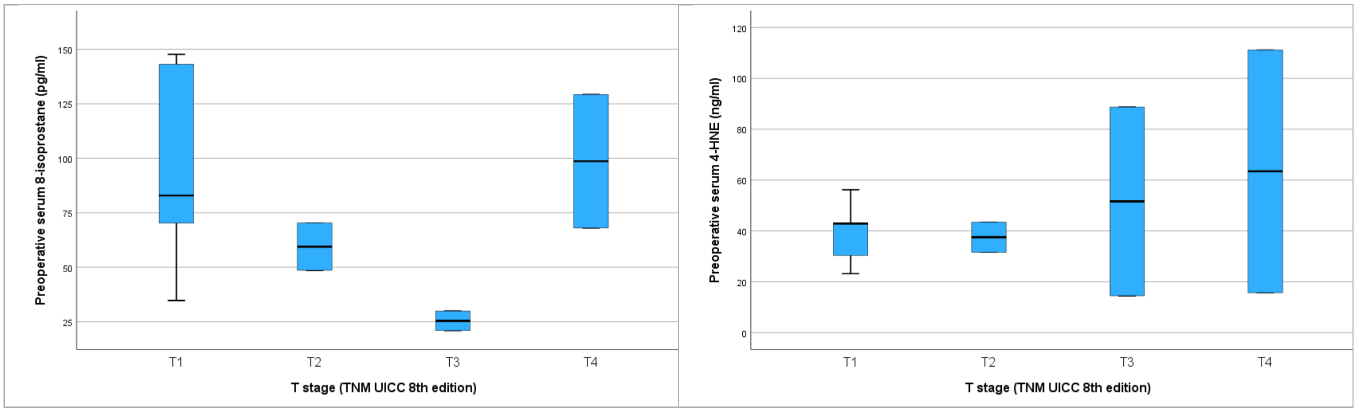

Supplement: Supplementary file 1 [file antioxidants-13-00590-s001.zip › antioxidants-2974774-supplementary.pdf]
